# Maternal knowledge about long-term consequences of pregnancy complications – a cross-sectional study

**DOI:** 10.1186/s12884-025-08156-0

**Published:** 2025-09-18

**Authors:** Marlene Böhm, Constantin von Kaisenberg, Cordula Schippert, Frauke von Versen-Höynck

**Affiliations:** Department of Obstetrics and Gynecology, Hannover Medical School, Hannover, Lower Saxony Germany

**Keywords:** Pregnancy complication, Preeclampsia, Preterm birth, Cardiovascular risk, Educational intervention, Knowledge

## Abstract

**Purpose:**

Pregnancy complications such as preeclampsia, obesity, and gestational diabetes mellitus are associated with an increased risk of cardiovascular diseases (CVD) later in life. This study aimed to evaluate mothers’ knowledge about these complications, their potential long-term consequences, and the education they received regarding cardiovascular risks.

**Methods:**

This cross-sectional study involved mothers who gave birth at a tertiary academic hospital. Participants completed an online questionnaire, and frequencies of demographic data, pregnancy-related information, and knowledge about long-term risks were calculated. Knowledge was categorized using Bloom’s cut-off based on responses to 25 questions regarding CVD risk factors, and associations between Bloom’s knowledge categories and various independent categorical variables were analyzed using the Pearson Chi-squared test, with a significance level set at *p* < 0.05.

**Results:**

From May to August 2024, 774 women were contacted, with 144 responses included in the analysis. The mean age of participants was 36 ± 4.9 years. Over half reported moderate knowledge of cardiovascular risk factors. Notably, 73% were unaware of the increased risk of CVD following pregnancy complications, 72% lacked knowledge of potential long-term adverse outcomes, and 73% did not receive educational interventions regarding long-term risks upon hospital discharge.

**Conclusion:**

The study identified a significant gap in women’s understanding of the increased CVD risks associated with pregnancy complications. Furthermore, the educational interventions provided at discharge were either lacking, insufficiently detailed, or not memorable enough for mothers to retain essential information.

**Supplementary Information:**

The online version contains supplementary material available at 10.1186/s12884-025-08156-0.

## Introduction

Cardiovascular diseases (CVD) remain globally the leading cause of death for both women and men [1]. While the incidence of CVD has declined in older populations, it has stagnated among younger individuals, particularly women of reproductive age [2, 3]. The risk of developing cardiovascular conditions is shaped by a multitude of factors, including lifestyle and pre-existing conditions, such as pregnancy complications [4, 5].

Adverse pregnancy outcomes – including gestational diabetes [6], gestational hypertension [7], preeclampsia [8, 9] (including eclampsia [10]), preterm birth [11] and placental abruption [12] – have been linked to an increased long-term risk of cardiovascular and metabolic diseases [13]. Specifically, conditions such as arteriosclerotic CVD (including coronary heart disease, peripheral vascular disease, and ischemic stroke), heart failure, and hemorrhagic stroke are strongly associated with adverse pregnancy outcomes earlier in life [14].

While some pregnancy complications are relatively rare, others are more common. Gestational diabetes affects approximately 14% of pregnancies worldwide [15], while gestational hypertension occurs in 5–10% of pregnancies with half of these cases progressing to preeclampsia [16, 17]. Preeclampsia affects 4.6% of pregnancies, while eclampsia occurs in 1.4% of births [18]. Placental abruption, although less frequent, affects 0.4–1.5% of pregnancies, and stillbirths occur in about 1.4% of births globally [19]. Preterm birth (< 37 weeks) impacts nearly 9.9% of births worldwide [20].

The mechanisms underlying the development of long-term cardiovascular risks following adverse pregnancy outcomes, such as preeclampsia, are not fully understood [21]. However, it is well-established that these complications cause significant cardiovascular damage during pregnancy. While many symptoms resolve postpartum, the elevated risk profile often persists. For instance, women with gestational hypertension have a 2-4-fold increased risk of developing chronic hypertension later in life [22]. Preeclampsia doubles the risk of mortality from CVD [21], and gestational diabetes increases the risk of CVD by twofold, independent of progression to type 2 diabetes mellitus [6]. Additionally, women with gestational diabetes face a 7-fold higher risk of developing type 2 diabetes mellitus later in life [23].

Despite the strong evidence linking adverse pregnancy outcomes to long-term cardiovascular risks, significant gaps remain in the awareness, education, and follow-up care for affected women. Studies reveal disparities in CVD assessment between women and men, with women often receiving less comprehensive evaluation and care [24, 25]. Factors, such as poor awareness, inadequate follow-up, and barriers to lifestyle change (e.g., cost of healthy food) contribute to low participation in preventive programs [26–28]. Women’s knowledge plays a critical role in post-pregnancy cardiovascular monitoring (e.g., blood pressure tracking, lifestyle adjustment, and diet) and in empowering self-management of symptoms [29]. Early educational interventions could promote healthier behaviors and reduce risks for both mother and child [30].

The objective of this study was to evaluate the current level of knowledge among women who have experienced adverse pregnancy outcomes related to long-term cardiovascular and metabolic consequences, as well as to identify the factors that influence their understanding of these health implications.

## Methods

### Study setting, design and participants

This cross-sectional, anonymous study was conducted at the Department of Obstetrics and Gynecology at Hannover Medical School (MHH), a tertiary care academic hospital in Germany.

We applied two inclusion criteria: women were eligible to participate if they had given birth between January 2020 and August 2023 and had experienced one or more through ICD-codes defined complications during their pregnancy. Potential participants were identified through the electronic medical record system using ICD-codes for conditions such as gestational hypertension (O13), gestational diabetes (O24.4, O24.9), preeclampsia (O14), eclampsia (O15), fetal growth restriction (O36.5), placental abruption (O45), or preterm delivery (O60.1, O60.3), resulting in a total of 774 women. Women who gave birth in another time frame and women without the listed complications were excluded from the study.

Women were contacted via mail, which included a written invitation to participate in the study and a QR-Code linking to a specially developed online questionnaire. The online questionnaire remained accessible from May 8th to August 10th, 2024.

### Questionnaire

The questionnaire was developed based on existing, evaluated questionnaires from other studies with certain questions added and modified to suit our research objectives [1, 2, 31–38]. The survey was tested with 37 participants, and their feedback was incorporated into the final version. The questionnaire was hosted online on the SoSciSurvey platform and was available exclusively in German, with an English version provided in the supplementary materials. The questionnaire was organized into four distinct sections.

The first section collected general sociodemographic data, including the age of the participants and their child, Body-Mass-Index (BMI), education level and employment status.

The second section focused on the participant’s pregnancy history. Questions included the number of pregnancies, outcomes of those pregnancies, and the presence of complications such as gestational hypertension, preeclampsia, eclampsia, gestational diabetes, placental abruption, and preterm birth. Participants were also asked about the timing and reasons for preterm delivery, including the gestational age at the time of birth, as well as any hospitalization during pregnancy.

The third section assessed participants’ knowledge of pregnancy complications and their long-term health implications. Questions focused on general knowledge of CVD risk associated with these complications, awareness of potential long-term consequences, and the participant’s perception of their own CVD-risk. Additionally, participants were asked if they felt capable of explaining their pregnancy complication to others and whether they knew how to improve their health for future pregnancies. They were also prompted to evaluate their understanding of their personal CVD risk.

At the end of this section, participants responded to 25 statements regarding general CVD risk factors (e.g., symptoms of a heart attack, risk factors such as high cholesterol and obesity) by selecting “true”, “false” or “I don’t know”. These responses were used to calculate Bloom’s cut-off.

The fourth section examined whether participants had received information about CVD risks and the long-term implications of their pregnancy complication(s) at the time of hospital discharge. For those who reported receiving information, they were asked to identify the source. However, the final question regarding the source of information was excluded from the analysis due to inconsistencies in the data, the number of participants who reported being informed at discharge did not match the number who specified the source of that information.

### Data analysis

Three criteria were defined to determine the inclusion of participants’ data in the analysis. First, participants were required to provide online consent to participate in the survey, agree to the privacy policy, and accept the declaration of consent. Second, they needed to complete the questionnaire by reaching the final page. Lastly, participants were required to answer more than 50% of the questionnaire items. The results are based on the number of responses from the participants. Those who met the inclusion criteria but did not answer a specific question of the questionnaire were excluded from the statistical analysis for that particular question. As an initial step, the frequencies of the demographic data, pregnancy-related information, participant’s knowledge about the long-term risks, and the information provided at hospital discharge were calculated. Time of birth and weight were divided into common categories [39].

To quantify participant’s knowledge, Bloom’s cut-off was determined based on the responses to the 25 questions regarding general risk factors for CVD. All participants were required to answer these questions. Bloom’s cut-off categorizes knowledge into three levels: poor, moderate, and high. Knowledge was classified as high if participants answered at least 80% of the questions correctly, moderate for 60–79% correct answers, and poor for less than 60% correct responses [40].

Questions allowing multiple answers (e.g., selection of pregnancy complications, reasons for hospitalization during pregnancy, possible long-term consequences of pregnancy complications, or the individual providing information at discharge) were summarized into sets of responses.

The Pearson Chi-squared test for homogeneity was employed to analyze associations between variables, with cross-tabulations utilized to explore relationships. Specifically, we examined the association between Bloom’s cut-off knowledge categories and independent categorial variables, including self-assessed CVD risk, knowledge about optimizing pregnancy outcomes, ability to explain pregnancy complications, receipt of health risk information, and level of secondary education. A p-value of less than 0.05 was considered statistically significant. Data analysis was performed using IBM SPSS Statistics Version 29.0.2.0.

During our analysis, we discovered inconsistencies between the documented ICD-codes and the responses in the questionnaires related to pregnancy complications. To further investigate these discrepancies, we cross-referenced the data with information from the participants’ hospital discharge letters. We then calculated Cohen’s kappa to evaluate the level of agreement between the questionnaire responses and the discharge letters and ranges from “slight agreement” (0–20%) to “almost perfect agreement” (> 80%) [41].

## Results

**Fig. 1:**
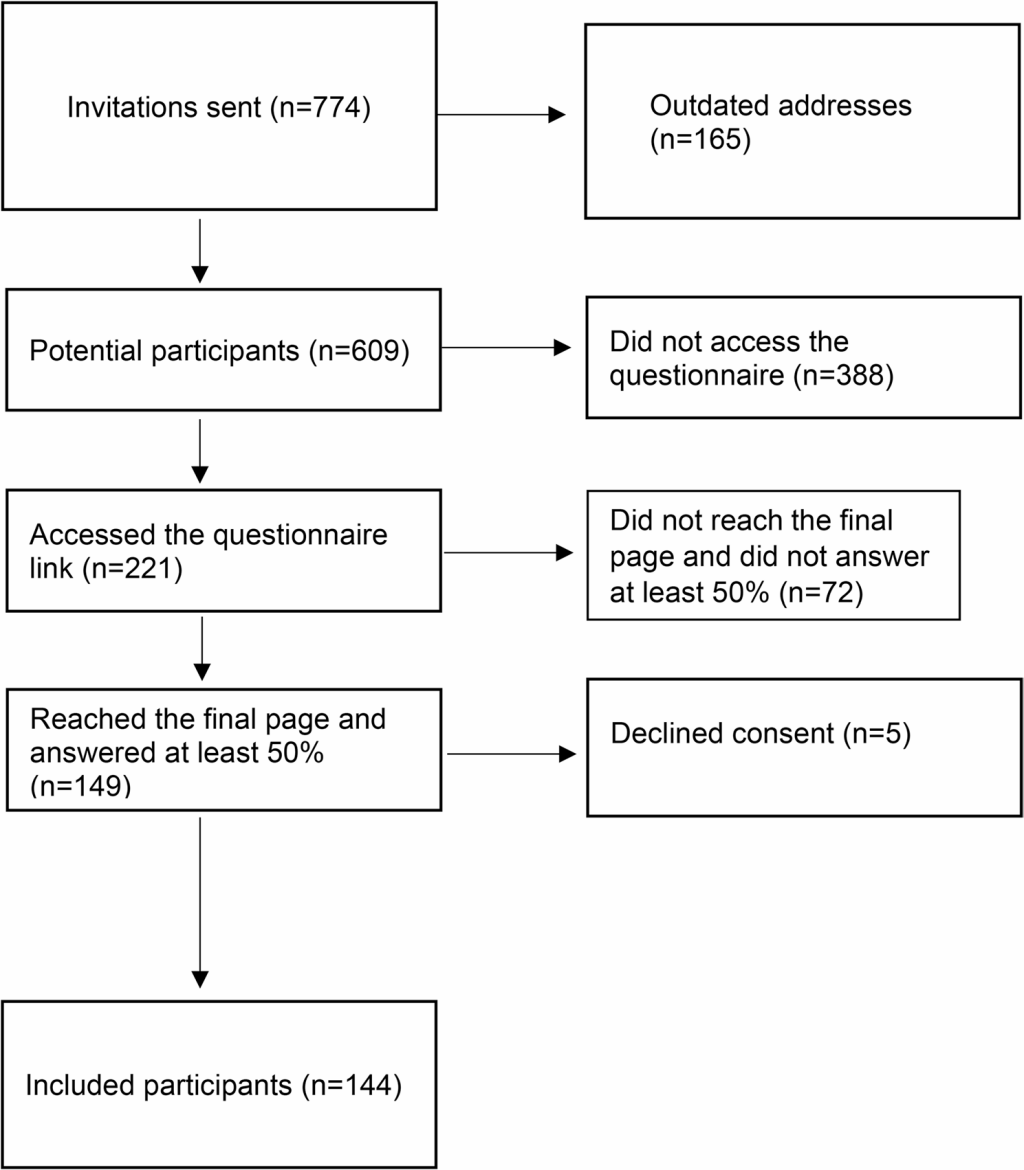
Participant recruitment flowchart

Out of the 774 invitations sent, 165 were returned due to outdated addresses. The questionnaire link was accessed a total of 221 times. Among these, 178 participants completed at least parts of the questionnaire, while 149 participants reached the final page and answered at least 50% of the questions. Five participants declined to provide consent for participation and data processing. As a result, data from 144 participants were included in the final analysis (Fig. 1).

The overall response rate, defined as the proportion of participants who completed the questionnaire, was 29%, while the attrition rate, representing those who started but did not finish the questionnaire, was 18%.

### Sociodemographic information

The baseline characteristics of the study population are summarized in Table 1. The mean age of participants was 36 ± 4.9 years. Most of the women were highly educated, with 77% holding at least a high school diploma. Furthermore, more than half (54%) had completed tertiary education, and 65% were engaged in gainful employment (65%).

**Table 1:** Sociodemographic information of study participants

Baseline characteristics	Outcome data n (%)
Age categories, years (*n=*144)	*n=***144**
<30	14 (10)
30-35	43 (30)
35-40	62 (43)
>40	25 (17)
BMI-categories (*n=*132)	*n=***132**
Underweight	1 (0.8)
Normal weight	68 (52)
Overweight	26 (20)
Obesity grade 1	15 (11)
Obesity grade 2	11 (8)
Obesity grade 3	11 (8)
Secondary education (*n=*144)	*n=***144**
Secondary school certificate (8^th^ grade)	1 (0.7)
Secondary school certificate (10^th^ grade)	29 (20)
Highschool graduate	111 (77)
Others	3 (2)
Tertiary education completed (*n=*144)	*n=***144**
Yes	139 (97)
No	5 (4)
Gainful employment (*n=*144)	*n=***144**
Yes	93 (65)
No	51 (35)
Live births (*n=*139)	*n=***139**
0	1 (0.7)
1	50 (36)
2	61 (44)
3	19 (14)
4	6 (4)
5	2 (1)
Miscarriages (*n=* 102)	*n=***102**
0	58 (57)
1	34 (33)
2	6 (6)
3	3 (3)
8	1 (1)
Stillbirths (*n=*78)	*n=***78**
0	72 (92)
1	6 (8)
Abortions (*n=*83)	*n=***83**
0	73 (89)
1	8 (10)
2	2 (2)
Ectopic pregnancies (*n=*77)	*n=***77**
0	74 (96)
1	3 (4)

In terms of BMI, 52% of participants reported a normal weight, while 20% were classified as overweight, and 28% had obesity grades 1–3 at the time of completing the survey. Nearly all participants (99%) reported having experienced at least one live birth. However, a significant proportion also reported adverse pregnancy outcomes, including one or more miscarriages (43%), stillbirths (8%), induced abortions (12%), and ectopic pregnancies (4%), in addition to the index pregnancy.

### History of the index pregnancy

Detailed information on the index pregnancies of the study population is provided in Table 2. More than half of the participants (68%) reported experiencing a pregnancy complication, including gestational hypertension, preeclampsia, eclampsia, gestational diabetes, placental abruption, or preterm birth, while 32% reported none of these complications. The most frequently reported complications were gestational diabetes (31%), preterm birth (27%), and gestational hypertension (16%).

**Table 2:** Information about the index pregnancy

Baseline characteristics	Outcome data n (%)
Pregnancy complications* (*n=*144)	*n=***144**
Gestational hypertension	23 (16)
Preeclampsia	18 (13)
Eclampsia	2 (1)
Gestational diabetes	44 (31)
Placental abruption	1 (1
Preterm birth	38 (26)
None of the given answers	46 (32)
Preterm birth (*n=*142)	*n=***142**
Induced preterm birth	7 (5)
Cesarean section before start of contractions	28 (20)
Spontaneous preterm birth	19 (13)
No preterm birth	88 (62)
Gestational age at delivery (*n=*143)	*n=***143**
Extreme prematurity (≤ 28 weeks of gestation)	4 (3)
Early preterm birth (≤ 32 weeks of gestation)	7 (5)
Moderate preterm birth (≤ 34 weeks of gestation)	8 (6)
Late preterm birth (≤ 37 weeks of gestation)	31 (22)
Term pregnancy (≤ 42 weeks of gestation)	93 (65)
Reasons for hospitalization during pregnancy (*n=*144) *	*n=***144**
Gestational hypertension	13 (9)
Preeclampsia	14 (10)
Eclampsia	1 (0.7)
Gestational diabetes	4 (3)
Placental abruption	1 (0.7)
Threatened preterm birth	8 (6)
Beginning of labor	5 (4)
Other reasons**	36 (25)
None of the provided answers	74 (51)

*) multiple answers possible (the percentage for these questions could exceed 100% reflecting the potential for multiple selections per participant), Data are shown as number (%)

**) birth induction, multiple pregnancy, other birth complications (e.g. cervical insufficiency, premature rupture of membranes, premature birth, hemorrhage, breech presentation, malformations, placental insufficiency, rhesus incompatibility), diseases not related to the birth

Regarding delivery outcomes, 62% of women had a term delivery, while 20% underwent a cesarean section before labor began. Additionally, 14% experienced spontaneous delivery before 37 weeks, and 5% had a medically induced preterm delivery. 65% of the participants delivered at term, while 35% experienced preterm births (Table 2).

Approximately half of the participants reported no hospitalization during their pregnancy. However, 10% were hospitalized due to preeclampsia, 9% for gestational hypertension, and 6% due to threatened preterm birth.

### Awareness of pregnancy complications and knowledge about long-term consequences

**Fig. 2:**
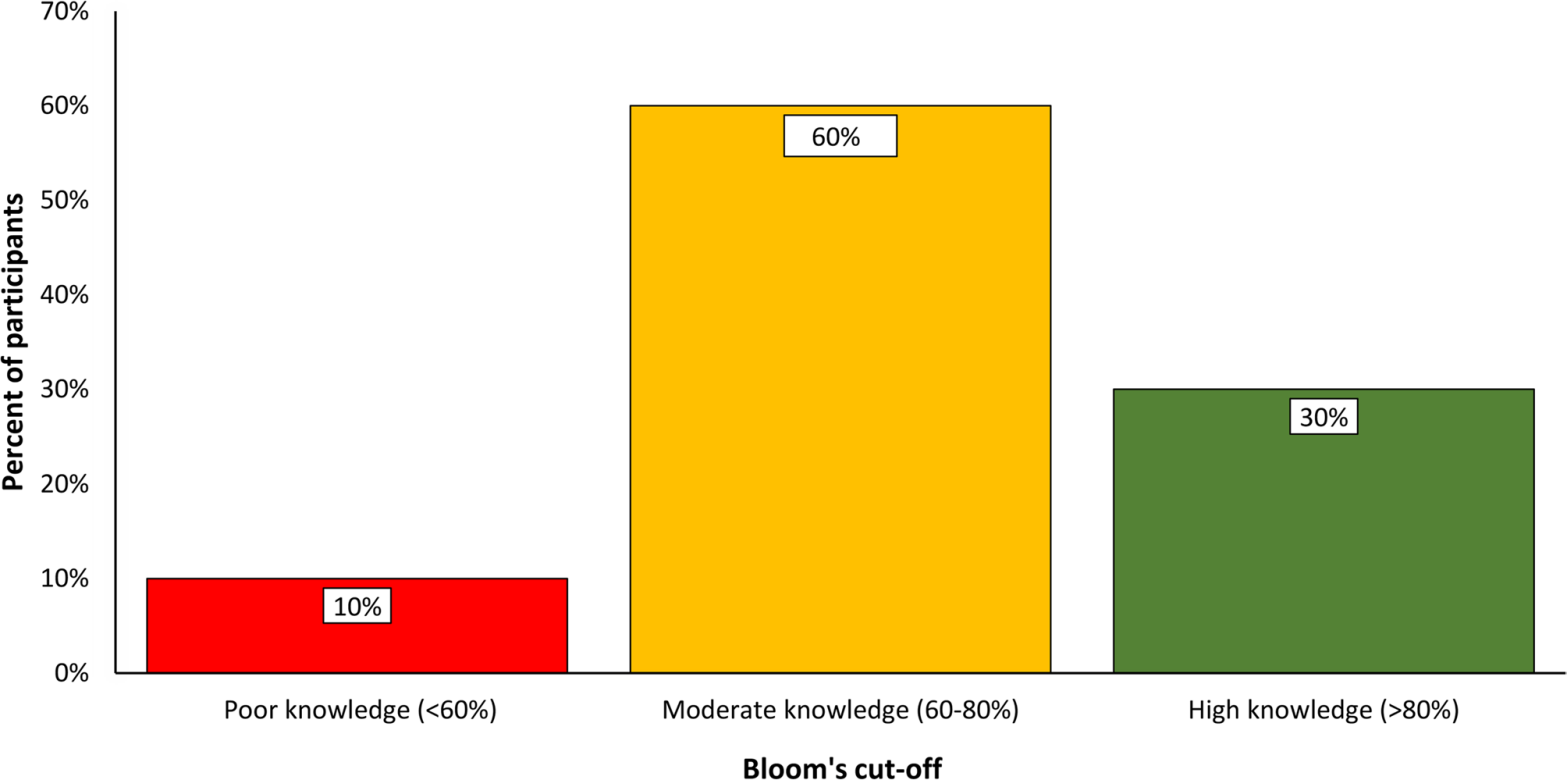
Distribution of bloom's cut-off levels indicating knowledge of CVD risk among study participants

Details of participant’s knowledge are summarized in Table 3. Knowledge about CVD risks was assessed using Bloom’s cut-off tool, revealing that 10% of participants had poor knowledge, 60% had moderate knowledge, and 30% demonstrated high knowledge regarding CVD risks (Fig. 2).

**Table 3:** Knowledge about long-term consequences of pregnancy complications

Baseline characteristics	Outcome data *n* (%)
Knowledge about CVD risk (*n=*141)	*n=***141**
Yes, I know, that the pregnancy complication is associated with an increased CVD risk	25 (18)
Yes, I know, that the pregnancy complication isn’t associated with an increased CVD risk	13 (9)
No, I didn’t know about the risk	103 (73)
Long-term consequences (*n=*144)	*n=***144**
Heart attack	8 (6)
Stroke	8 (6)
Hypertension	25 (18)
Heart failure	9 (6)
Hypertensive nephropathy	7 (5)
There are no long-term consequences	103 (72)
Capability to explain the pregnancy complication (*n=*136)	*n=***136**
Yes	98 (72)
No	13 (10)
I don’t know	25 (18)
Knowledge about optimizing health for a future pregnancy (*n=*137)	*n=***137**
Yes	78 (57)
No	40 (29)
I don’t know	19 (14)
Self-assessed risk of personal CVD (*n=*140)	*n=***140**
Much higher than average	15 (11)
Higher than average	46 (33)
Average	60 (43)
Lower than average	10 (7)
Much lower than average	9 (6)
Knowledge about how to reduce the CVD risks (*n=*140)	*n=***140**
Very well informed	24 (17)
Well informed	46 (33)
Moderately informed	40 (29)
A little informed	13 (9)
Not informed	17 (12)
Information about CVD risk received at discharge (*n=*142)	*n=***142**
Yes	28 (20)
No	103 (73)
I don’t know	11 (8)

Nearly three quarters of participants (73%) were unaware of the increased risk of CVD following a pregnancy complication, while 18% recognized this association. Regarding long-term consequences of pregnancy complications, 72% reported no knowledge of potential adverse outcomes. Among those who identified consequences, the most commonly reported were hypertension (18%), followed by heart failure (6%), heart attack (6%) and stroke (6%).

When asked about their ability to explain their pregnancy complication, 72% of participants felt capable, while 10% did not. The majority (57%) of participants with poor knowledge were unsure or felt incapable of explaining their pregnancy complication. Conversely, 93% of the participants with high knowledge reported feeling confident in explaining their pregnancy complication to others.

A significant association was observed between Bloom’s cut-off and participant’s ability to explain their pregnancy complication to others (*p* = 0.004), as summarized in Table 4. Over half of the participants (57%) reported knowing how to optimize their health before a subsequent pregnancy, while 29% stated they did not know. Participants with poor knowledge were significantly less likely to know how to improve their health conditions, with 64% of this group expressing uncertainty or lack of awareness. In contrast, 70% of participants with high knowledge reported knowing how to optimize their health for a future pregnancy (Table 4).

**Table 4:** Analysis of factors influencing knowledge of cardiovascular risk (Bloom’s cut-off)

Variables		Bloom’s cut-off			p-value
		Poor knowledge	Moderate knowledge	High knowledge	
	*n* (%)	*n* (%)	*n* (%)	*n* (%)	
Assessment of personal CVD risks	*n=***140**	*n=***14**	*n=***85**	*n=***41**	0.02
Much higher than average	15 (11)	1 (7)	8 (9)	6 (15)	
Higher than average	46 (32)	1 (7)	32 (38)	13 (32)	
On average	60 (43)	11 (79)	37 (44)	12 (29)	
Lower than average	10 (7)	0 (0)	3 (4)	7 (17)	
Much lower than average	9 (6)	1 (7)	5 (6)	3 (7)	
Knowledge about optimizing a future pregnancy*	*n=***118**	*n=***11**	*n=***70**	*n=***37**	0.21
Yes	78 (57)	5 (36)	45 (54)	28 (70)	
No	40 (29)	6 (43)	25 (30)	9 (23)	
Explain the pregnancy complication to others*	*n=***11**	*n=***9**	*n=***64**	*n=***38**	0.004
Yes	98 (72)	6 (43)	55 (67)	37 (93)	
No	13 (10)	3 (21)	9 (11)	1 (3)	
Information on the health risks*	*n=***131**	*n=***14**	*n=***77**	*n=***40**	0.92
Yes	28 (20)	3 (20)	17 (20)	8 (19)	
No	103 (73)	11 (73)	60 (71)	32 (76)	
Secondary education	*n=***144**	*n=***15**	*n=***86**	*n=***43**	0.16
Secondary school certificate (8^th^ grade)	1 (0.7)	0 (0)	1 (1)	0 (0)	
Secondary school certificate (10^th^ grade)	29 (20)	6 (40)	19 (22)	4 (9)	
Highschool graduate	111 (77)	9 (60)	65 (76)	37 (86)	
Others	3 (2)	0 (0)	1 (1)	2 (5)	

*) minus the „I don’t know” category

Nearly half of the participants (43%) estimated their personal CVD risk as average, while 44% perceived it as higher than average, and 21% as lower than average. Most participants (79%) with poor knowledge assessed their personal CVD risk as average. In contrast, only 29% of participants with high knowledge rated their CVD risk as average, while 46% of this group estimating their risk as higher than average (Table 4). A significant association was observed between Bloom’s cut-off levels and participant’s assessment of their personal CVD risks (*p* = 0.017).

### Postpartum counseling and information

Half of the participants reported feeling well or very well informed about how to reduce their CVD risks, while the other half felt only moderately, slightly, or not at all informed.

Nearly three-quarter (73%) of the participants indicated that they received no educational intervention regarding the long-term risks associated with pregnancy complications upon discharge from the hospital, while 20% reported having received such information. The receipt of health risks information did not appear to significantly influence participant´s knowledge levels (*p* > 0.05).

The analysis also revealed a trend indicating that women with higher levels of education were more likely to demonstrate high knowledge (86%), whereas less educated participants were more likely to have moderate (22%) or poor knowledge (40%), (*p* = 0.16) (Table 4).

### Data consistency analysis

We identified inconsistencies in the data regarding pregnancy complications. Among the women with documented pregnancy complications in the hospital’s electronic medical record system, 32% reported no complications or none of the specified conditions. Cross-referencing this data with information from their hospital discharge letters revealed that only 23% of participants reported none of the pregnancy complications mentioned in the questionnaire. The analysis showed a 64% agreement between the questionnaire responses and the discharge letters, indicating a moderate level of inter-rater agreement in our study population, as defined by Landis and Koch [41].

## Discussion

This study evaluated women’s awareness of pregnancy complications, their long-term health consequences, and their knowledge of CVD risk covering a wide range of conditions beyond the commonly studied preeclampsia. The results were also analyzed in relation to sociodemographic factors, e.g. education. Our findings reveal notable gaps in knowledge, highlighting the need for improved postnatal education and targeted interventions to increase awareness of long-term health risks associated with pregnancy complications.

To our knowledge, this is the first study conducted in Germany to assess awareness of long-term risks following pregnancy complications among a cohort of young mothers. The sociodemographic characteristics of the study population indicate that participants were highly educated, with 97% having completed tertiary education—significantly higher than the German national average of 65% [41]. This educational profile may have influenced the knowledge levels observed in the study.

The prevalence of overweight (20%) and obesity (28%) in our study population is consistent with prior research on German women. In 2021 The Federal Statistical Office reported that 43% of German women were overweight and 15% were obese (https://de.statista.com/statistik/daten/studie/233461/umfrage/entwicklung-von-uebergewicht-und-adipositas-in-deutschland-unter-frauen/). While our study found a slightly lower prevalence of overweight individuals, it observed a higher rate of obesity, which may be explained by the focus on women with pregnancy complications.

A striking finding was the high prevalence of gestational diabetes (31%) compared to the national average of 9% in Germany [42]. This discrepancy is likely due to our inclusion of only women with documented pregnancy complications from a tertiary care academic hospital. More than half (68%) of participants reported experiencing at least one pregnancy complication, with gestational diabetes, preterm birth, and hypertensive disorders being the most common. Additionally, nearly half (50%) of the participants required hospitalization during pregnancy, primarily due to hypertensive disorders such as preeclampsia (10%) and gestational hypertension (9%). These findings underscore the significant burden of pregnancy-related health issues in this population and their potential long-term consequences.

A major concern identified in this study is the lack of awareness regarding the long-term health risks associated with pregnancy complications. While 30% of participants demonstrated high knowledge using Bloom’s cut-off, the majority (70%) had only moderate or poor knowledge about CVD risks. Alarmingly, only 18% of participants recognized the association between pregnancy complications and CVD, while nearly 73% were unaware of this connection. This aligns with previous research highlighting inadequate postnatal education and gaps in practitioners’ awareness of elevated CVD risks [43, 44].

Interestingly, participants tended to overestimate their knowledge. Despite the low awareness of long-term complications, nearly three-quarters (72%) felt confident in explaining their pregnancy complications, and over half (57%) reported knowing how to optimize their health for future pregnancies. This discrepancy may stem from the participants’ high education levels or from self-directed learning after experiencing pregnancy complications [45].

One of the most concerning findings is the lack of structured postpartum education. Only 20% of participants recalled receiving an educational intervention at hospital discharge. This aligns with previous studies showing that postnatal education is often insufficient and that healthcare providers are not adequately emphasizing the long-term risks of pregnancy complications [43, 44, 46].

Surprisingly, no significant association was found between receiving an educational intervention and knowledge levels. This contrasts with other studies demonstrating that postnatal education can effectively improve women’s understanding of pregnancy complications [47, 48]. One possible explanation is that the information provided at discharge was not detailed or memorable enough. At least 8% of participants could not even recall whether they had received any educational intervention.

Despite the lack of structured education, 50% of participants felt well-informed about CVD risk reduction strategies, potentially due to self-education through the internet and social media. Prior research has shown that online resources are a popular source of health information among women [45]. However, reliance on self-directed learning may lead to misinformation or incomplete knowledge [49].

Practitioners’ awareness and practices regarding pregnancy complications and long-term risks appear to be highly variable. While some studies report that healthcare providers are unaware of the increased CVD risks associated with pregnancy complications [43], others indicate that they acknowledge the risks but lack knowledge about specific conditions such as gestational hypertension [50]. This variability may contribute to the insufficient postnatal education reported by participants.

Discrepancies observed in the reported pregnancy complications highlight potential inaccuracies in the documentation process. Cross-referencing with hospital discharge letters revealed that 23% had no reported complications, and the moderate inter-rater agreement (Cohen’s kappa = 0.64) suggests that while there is some consistency in the data, further investigation is warranted to understand the reasons behind these inconsistencies. The issue of miscoding or misclassification of participants may significantly impact the findings, particularly concerning their self-assessment of personal CVD risk. If participants are inaccurately classified regarding their pregnancy complications, it could lead to an underestimation or overestimation of their perceived risk, ultimately affecting the correlation between their knowledge of CVD and their self-assessment. Addressing these misclassifications is crucial for ensuring the validity of the study’s conclusions and for accurately interpreting the relationship between pregnancy complications and cardiovascular health. The study also faced challenges in participant recruitment. Approximately 21% of the intended population did not receive an invitation due to outdated addresses. Although email invitations may have been more successful, email addresses can also change over time and were not available to us. Also, there has not been a reminder for the participants to take part in the study. This would have potentially improved the response rate which is important for the validity of the study. In consultation with the Hannover Medical School’s Institute of Biometry we decided not to try to improve the response rate because of the retrospective and explorative study design.

Additionally, the use of a cross-sectional design limits our ability to assess changes in knowledge over time.

Another methodological limitation is the use of the chi-squared test in data samples with many zero-cells, which may impact the reliability of statistical associations. Thus, caution is warranted when interpreting these findings.

## Conclusions

This study highlights a critical gap in women’s knowledge about the long-term consequences of pregnancy complications, particularly their association with CVD risk. The findings suggest that current postnatal education efforts are insufficient, with only a minority of women receiving educational interventions at hospital discharge. Even when such interventions occur, they may not be detailed or impactful enough to improve knowledge retention.

To address these issues, healthcare providers should implement comprehensive educational interventions before hospital discharge, ensuring that women understand the long-term health risks associated with pregnancy complications. Practitioners must also be trained to recognize and communicate these risks effectively. Increased awareness among both patients and providers could empower women to take preventive measures, ultimately improving long-term health outcomes. A first step in the right direction was accomplished with the recently revised German S2k guideline for Hypertensive Disorders in Pregnancy: Diagnosis and Treatment, which updates the recommendations for postpartum care and includes a Postpartum Care Pass for Mothers After Preeclampsia [51]. Future research should explore the effectiveness of different educational approaches, including digital resources, in enhancing women’s knowledge and promoting cardiovascular health awareness.

## Supplementary Information

Below is the link to the electronic supplementary material.

Supplementary Material 1

## Data Availability

Data is included in the manuscript, and additional data can be obtained upon request from the corresponding author.
