# Supplementary material for: Maternal knowledge about long-term consequences of pregnancy complications – a cross-sectional study
Source: BMC Pregnancy Childbirth. 2025 Sep 18;25:935. doi: 10.1186/s12884-025-08156-0 (PMC12447609; doi:10.1186/s12884-025-08156-0)

Page 01

SD

1

. What is your study number?

Study number:

First, we want to ask you some general questions.

2

. When were you born?

Please add year, month and day of birth.

I was born

on

3

. Please state the date of birth of your child at the MHH

My child was

born on

4

. Please state your height and weight

Height:

Weight:

cm

kg


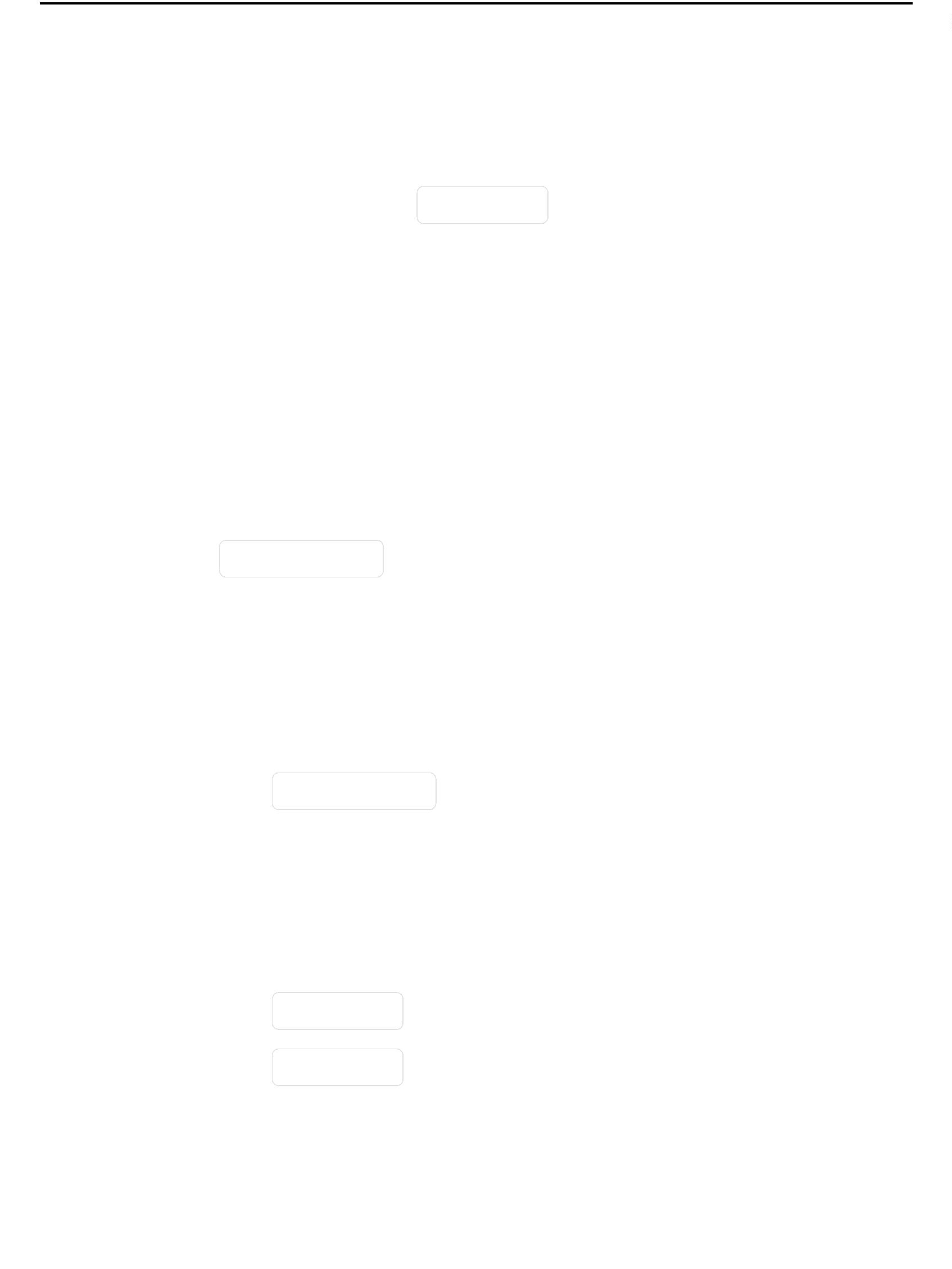


5

. What is your highest level of education?

Still in school

Finished school without a degree

Hauptschulabschluss (lower-level secondary education)

Realschulabschluss (mid-level secondary education)

Degree from polytechnic secondary school

Advanced technical college degree

Abitur (higher-level secondary education)

Others:

6

. What is your highest professional qualification?

No professional qualification

Vocational training period with final certificate, but no apprenticeship

Semi-skilled worker qualification

Completed commercial or agricultural apprenticeship

Completed business apprenticeship

Internship, traineeship

Vocational school qualification

Technical college degree

Master craftsman, technician or equivalent technical college qualification

Advanced technical college degree

University degree (Bachelor, Master, state examination)

PhD

Others:


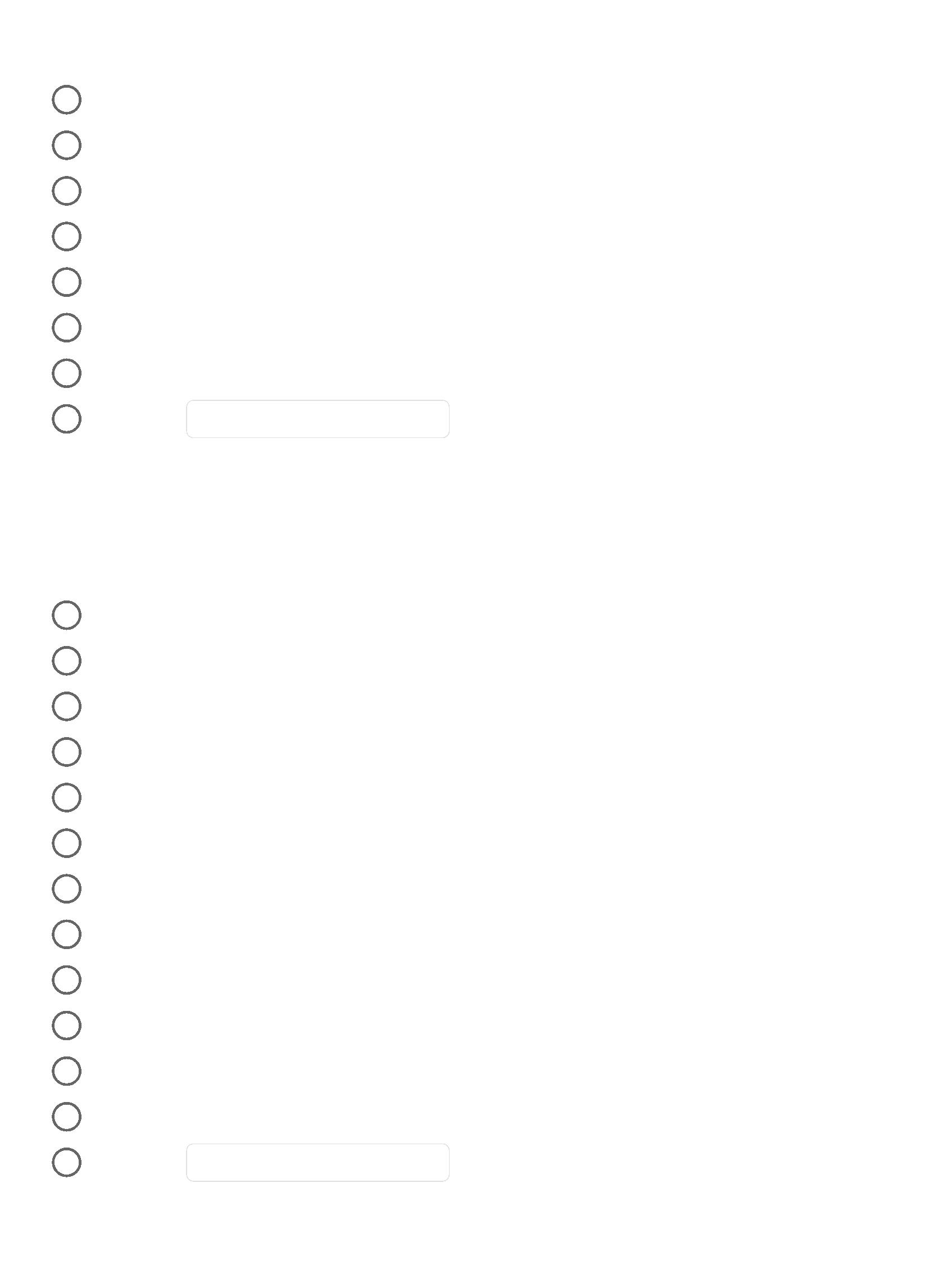


7

. What is your current profession?

Pupil

In training

Student

Employee

Public servant

Self-employed

Unemployed/seeking employment

Others:

8

. Are you currently employed?

Yes, I am employed.

No, I am unemployed.

No, I am in parental leave.

No, I am in maternity protection.

No, I am housewife.

No, I am pensioner.

No, I am student.

No, I am pupil.

No.


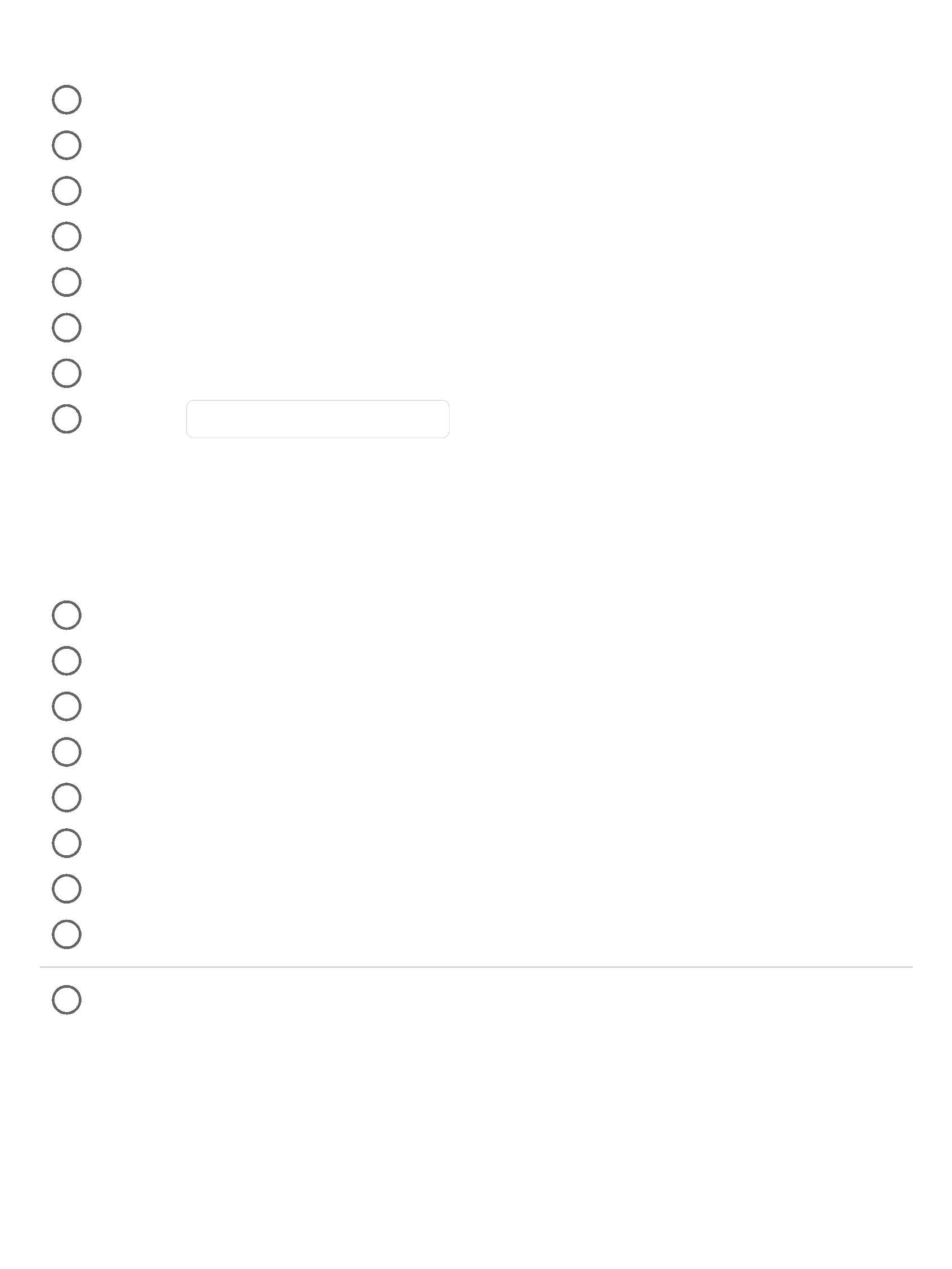


Page 04

SS

Pregnancy

Now we want to ask you more about your pregnancy.

9

. How often have you been pregnant until now?

Number of

pregnancies:

Of these were:

children born alive

miscarriages

extrauterine pregnancies

stillbirths

abortions

1

0. Did you have one of the following pregnancy complications?

Yes, I had gestational hypertension.

Yes, I had preeclampsia.

Yes, I had eclampsia.

Yes, I had gestational diabetes.

Yes, I had placental abruption.

Yes, I had preterm birth.

No, I had none of the pregnancy complications.


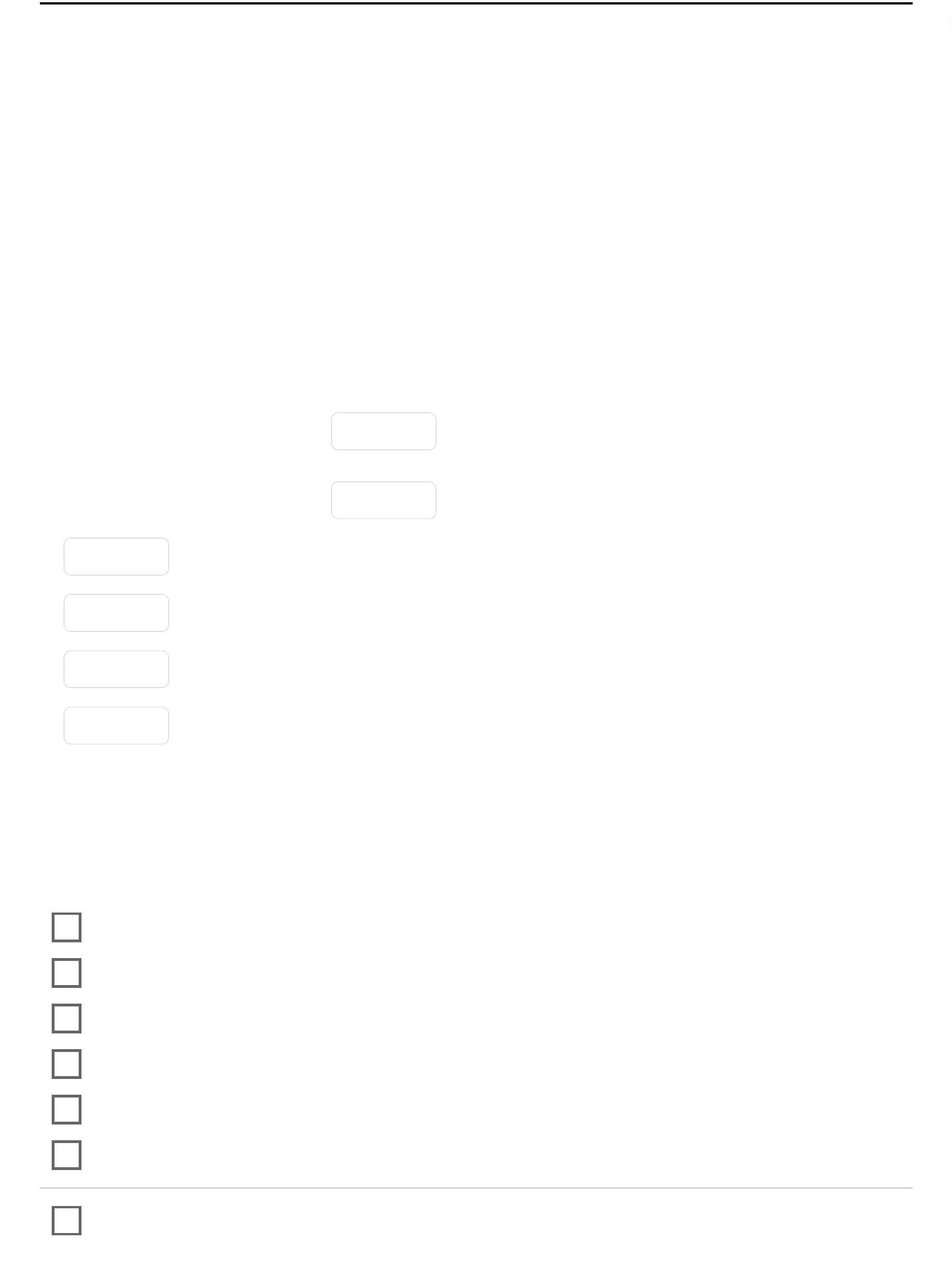


1

1. Did you give birth before the 37th week of pregnancy because of a

pregnancy complication?

Yes, the preterm birth was induced with medication.

Yes, I had a cesarean section before start of contractions.

Yes, I had a spontaneous preterm birth.

No, I did not give birth before the 37th week of pregnancy.

1

1

2. What week of pregnancy were you in when you gave birth?

Week of pregnancy

3. Have you been hospitalized during pregnancy?

Yes, because of gestational hypertension.

Yes, because of preeclampsia.

Yes, because of eclampsia.

Yes, because of gestational diabetes.

Yes, because of placental abruption.

Yes, because of a threatened preterm birth.

Yes, because of the beginning of birth.

Yes, because of:

No, I have not been hospitalized during pregnancy.


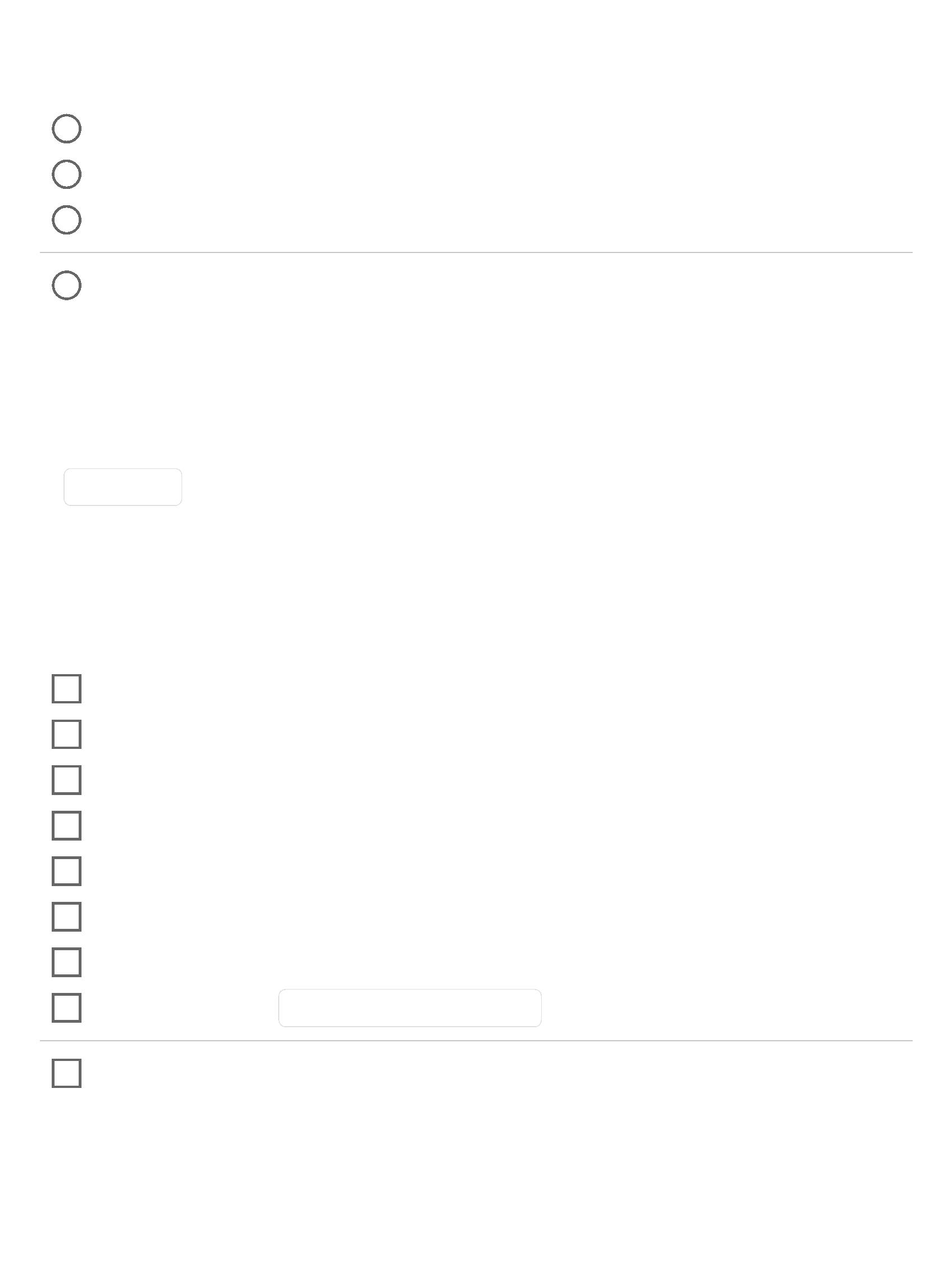


Page 06 CVD

We would then like to know what information you have about the possible

consequences of the pregnancy complication(s) you have experienced.

1

4. Do you know whether the pregnancy complications you experienced is/are

associated with an increased risk of cardiovascular disease in your future

life?

Yes, I know that the pregnancy complication(s) I experienced is/are associated with an increased risk of cardiovascular disease in my future life.

Yes, I know that the pregnancy complication(s) I experienced is/ are not

associated with an increased risk of cardiovascular disease in my future life.

No, I do not know anything about that.

1

5. What are some of the long-term risks of pregnancy complications?

Heart attack

Stroke

Hypertension

Hearth failure

Hypertensive nephropathy

There are no long-term risks of pregnancy complications.

1

6. Do you understand your pregnancy complication well enough to explain it

to another person?

Yes.

No.

I don’t know.

1

7. Do you know how to optimize the course of your next pregnancy?

Yes.

No.

I don’t know.

1

8. How would you assess your personal risk for cardiovascular diseases?

Much higher than average.

Higher than average.

On average.

Lower than average.

Much lower than average.

1

9. How would you rate your knowledge of how to reduce your personal

cardiovascular risk?

I am:

Very well informed.

Well informed.

Moderately informed.

A little informed.

Not informed.

The following part presents some statements, that you are asked to evaluate.

Please answer with "true", "false" or "I don’t know".

1

. Advanced age increases the risk to develop

True

True

False

False

I don’t

know.

coronary heart disease.

2

. Heart diseases caused by blockages of

I don’t

know.

coronary arteries develop over a long period of time and can easily stay undetected.

3

. Postmenopausal women have an increased

True

True

True

False

False

False

I don’t

know.

risk to develop heart diseases.

4

. An increased cholesterol level can cause

I don’t

know.

blockages of coronary arteries.

5

. Afro-American women have an increased risk

I don’t

know.

to develop heart diseases compered to white

women.

6

. High blood pressure can cause heart

True

True

True

False

False

False

I don’t

know.

diseases and strokes.

7

. Certain heart diseases can cause strokes.

I don’t

know.

8

. Symptoms of a stroke can be sudden

I don’t

know.

numbness and weakness of the face, arms,

legs, as well as sudden confusion.

9

. Smoking can cause blockages of coronary arteries.

True

False

I don’t

know.


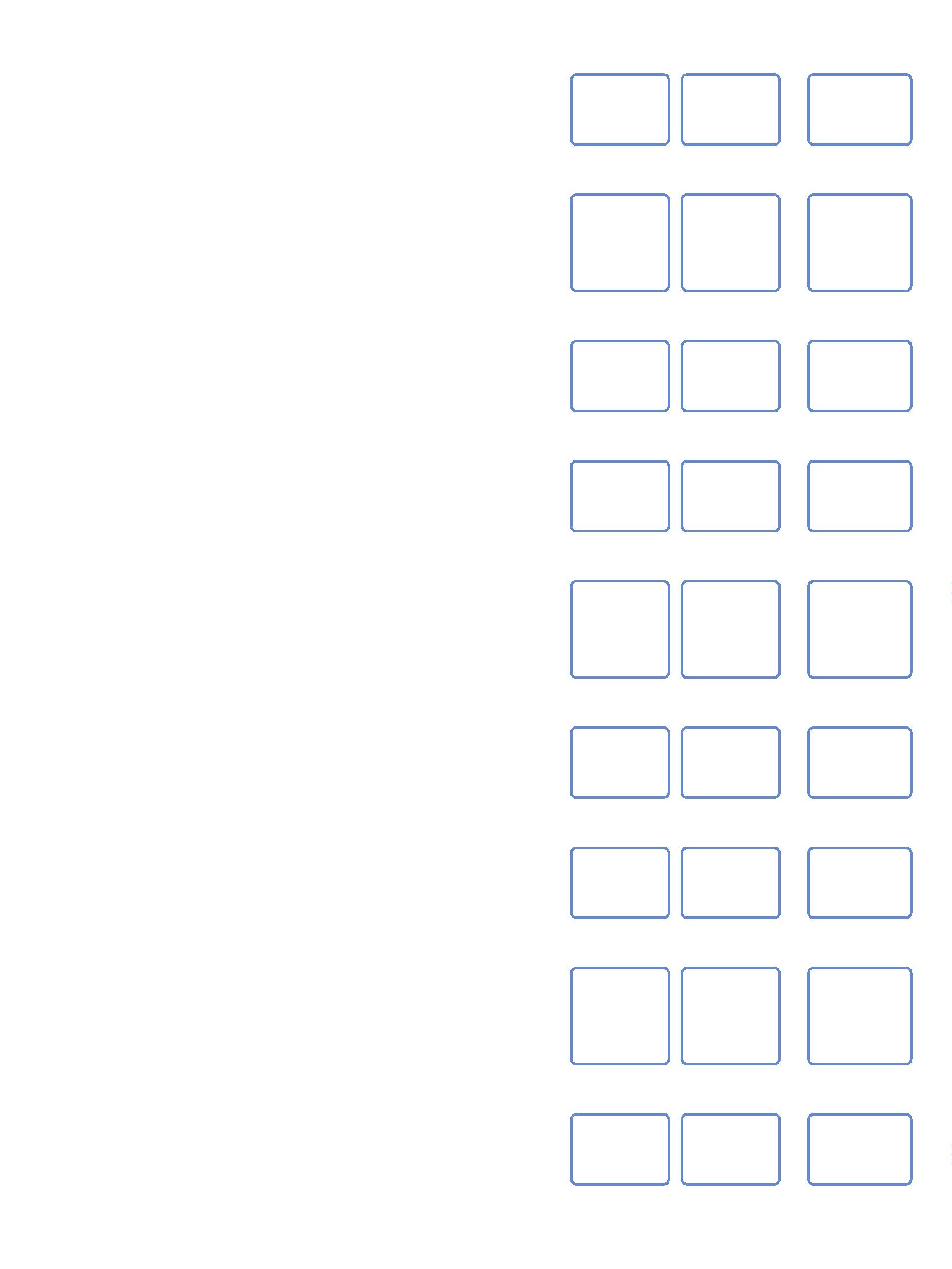


1

0. Chest pain, tightness of the chest and

True

False

I don’t

know.

unusual fatigue can be symptoms of a heart

attack.

1

1. Other symptoms of a heart attack can be

True

True

False

False

I don’t

know.

dyspnea, sweating and nausea.

1

2. Pain in the neck, shoulders, arms or back

I don’t

know.

and dizziness can be symptoms of a heart

attack.

1

3. Heart diseases and strokes are the most

True

True

True

True

True

True

True

False

False

False

False

False

False

False

I don’t

know.

common causes of death among women.

1

4. Genetic predisposition is a major risk

I don’t

know.

factor for heart diseases.

1

5. Less consumption of red meat can prevent

I don’t

know.

blockages of coronary arteries.

1

6. Nutritional cholesterol reduction can

I don’t

know.

prevent heart diseases.

1

7. Stress can elevate the risk for heart

I don’t

know.

diseases.

1

8. Obesity doesn’t influence the

I don’t

know.

cardiovascular risk.

1

9. There is evidence for hormone therapy to

I don’t

know.

prevent from heart diseases in women.


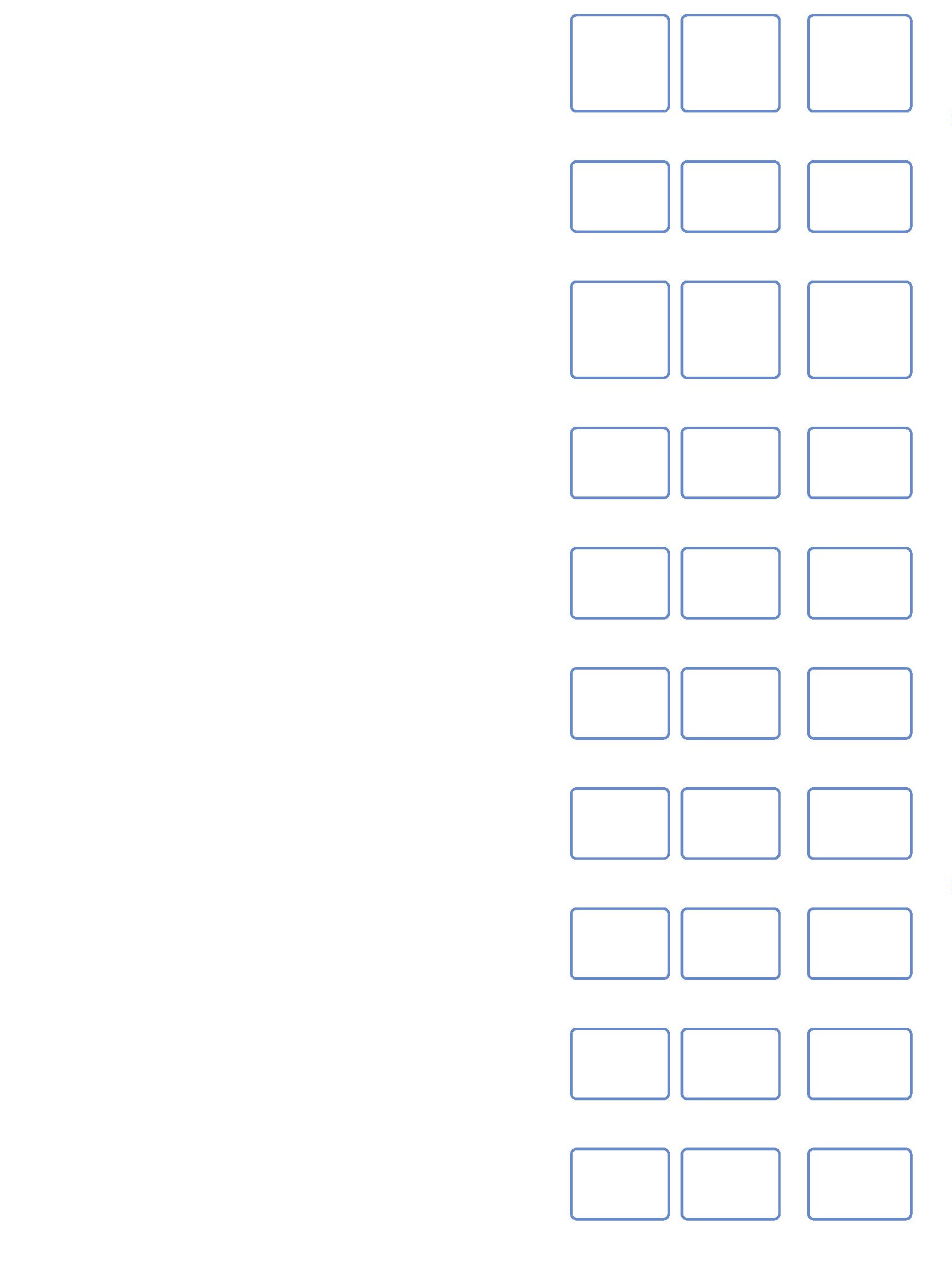


2

0. High-fat diet can cause blockages of coronary arteries.

True

True

True

True

True

True

False

False

False

False

False

False

I don’t

know.

2

1. Low female hormones can increase the risk

I don’t

know.

for blockages of coronary arteries.

2

2. Regular physical activity can prevent heart

I don’t

know.

diseases.

2

3. Diabetes can elevate the risk of heart

I don’t

know.

diseases.

24. A history of preeclampsia increases the

I don’t

know.

risk for heart diseases and stroke.

2

5. A familiar disposition of heart diseases

I don’t

know.

based on blockages of coronary arteries can

elevate the individual risk for heart diseases.

1


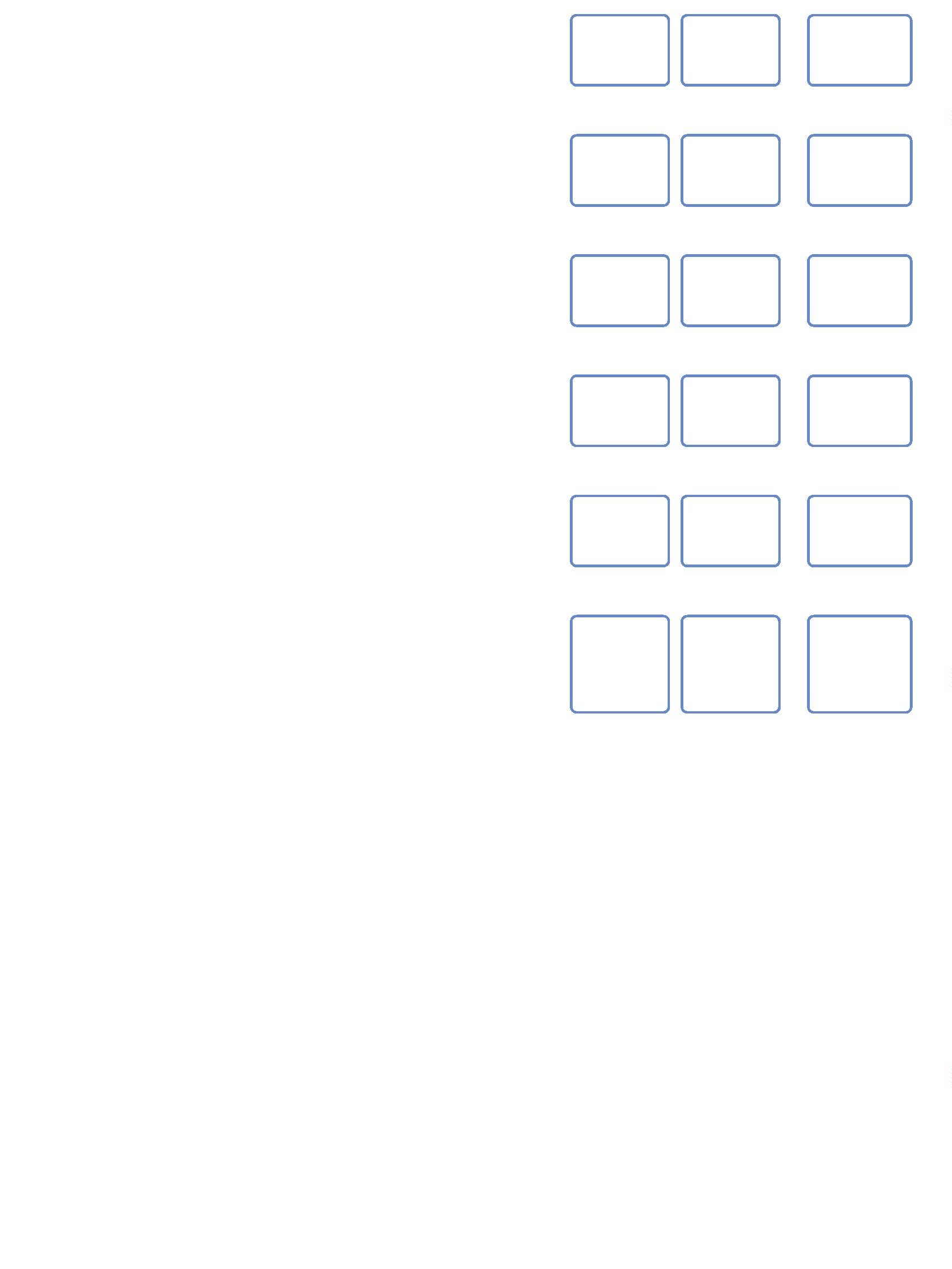


Page 12

AK

In the final section we would like to know more about your experiences during hospital discharge.

2

0. Have you received an information from medical stuff, midwifes, nurses or

physicians about possible complications and the health impacts on your future live while leaving the hospital?

Yes.

No.

I don’t know.

If yes:

2

1. Who gave you the information about possible long-term risks of your

pregnancy complication?

Midwife at the hospital

Nurses

Physician

Gynecologist in private practice

Midwife at home

Friends

I informed myself.

Another person:


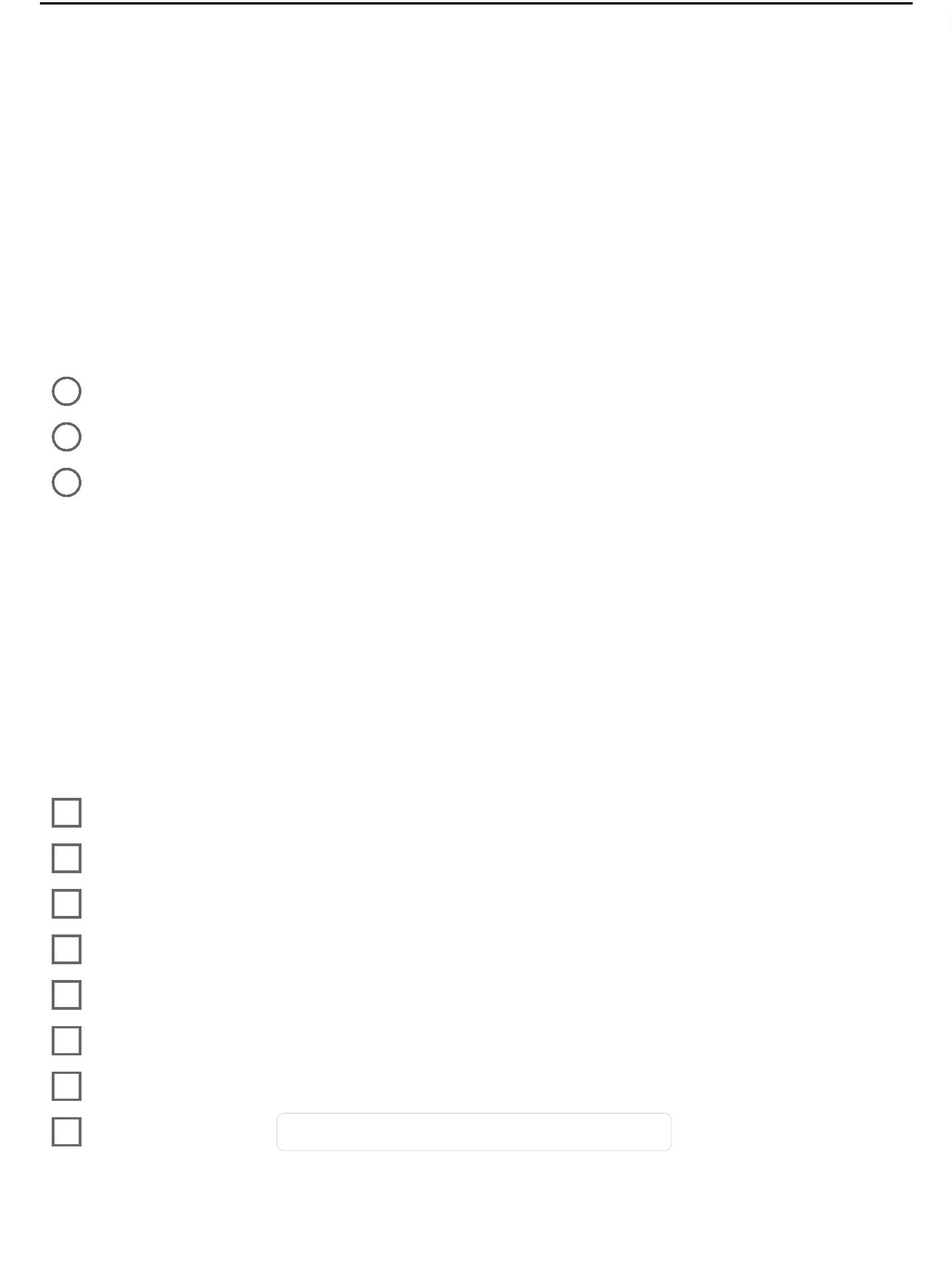

Supplement: Supplementary file 1 — Supplementary Material 1 [file 12884_2025_8156_MOESM1_ESM.docx]
